# Safety and Performance Evaluation of a Clinical Decision Support System for Imatinib Therapeutic Drug Monitoring: In Silico Study

**DOI:** 10.2196/93304

**Published:** 2026-10-21

**Authors:** Yuan James Pétermann, Roberto Rigamonti, Paul Thoueille, Junrui Chen, Lavinia Alberi, Alexandra Simon, François Veuve, Eva Choong, Bruno Da Rocha Carvalho, Sandro Carrara, François R Girardin, Thierry Buclin, Chantal Csajka, Yann Thoma, Monia Guidi

**Affiliations:** 1 Centre for Research and Innovation in Clinical Pharmaceutical Sciences, University Hospital of Lausanne and University of Lausanne, Rue du Bugnon 19, Lausanne, Vaud, 1005, Switzerland, 41 765437675; 2 Department of Clinical Pharmacology, University Hospital of Lausanne and University of Lausanne, Lausanne, Vaud, Switzerland; 3 School of Engineering and Management Vaud, HES-SO University of Applied Sciences and Arts Western Switzerland, Yverdon-les-Bains, Vaud, Switzerland; 4 Bio/CMOS Interfaces Lab, École Polytechnique Fédérale de Lausanne, Lausanne, Vaud, Switzerland; 5 VitalizeDx, Lausanne, Vaud, Switzerland; 6 Institute of Pharmaceutical Sciences of Western Switzerland, University of Geneva, University of Lausanne, Geneva & Lausanne, Switzerland

**Keywords:** therapeutic drug monitoring, clinical decision support system, oncology, model-informed precision dosing, population pharmacokinetic, point-of-care, imatinib, pharmacokinetics

## Abstract

**Background:**

Therapeutic drug monitoring (TDM) aims to optimize clinical outcomes but is hindered by logistical delays and susceptibility to data errors. Integrating model-informed precision dosing with point-of-care testing, fortified by rule-based validation and alerts, forms a transformative clinical decision support system (CDSS) essential for securing this complex workflow.

**Objective:**

This study evaluates, *in silico*, the effectiveness of a rule-based CDSS for imatinib in detecting data-quality issues and optimizing therapeutic dosing, compared with a human TDM expert.

**Methods:**

We simulated a set of 60 virtual patients using a previously developed population pharmacokinetic model and deliberately enriched it with data-quality errors (eg, timing inaccuracies and reporting errors) to stress-test system resilience. We compared CDSS and TDM expert performances based on their error-detection capabilities and the clinical impact on therapeutic target attainment and safety.

**Results:**

The CDSS and the expert demonstrated comparable error-detection capabilities. The CDSS showed numerically higher sensitivity (53%, 95% CI 35%‐70% vs 45%, 95% CI 30%‐61%) and lower specificity (83%, 95% CI 63%‐95% vs 100%, 95% CI 81%‐100%). Clinically, the CDSS demonstrated significantly higher rates of target attainment than the expert (33/60, 55% vs 22/60, 37%; *P*=.046). However, the CDSS resulted in 5 detrimental outcomes against 1 for the TDM expert, in which patients initially within the target range fell outside it.

**Conclusions:**

The CDSS demonstrated high efficacy in optimizing imatinib exposure while serving as a robust digital gatekeeper. While active optimization drives higher efficacy, a few observed detrimental cases highlight that algorithmic safety remains contingent on data integrity. This proof-of-concept study validates the CDSS architecture as a prerequisite for future prospective clinical trials.

## Introduction

Deeply rooted in the philosophy of precision medicine, therapeutic drug monitoring (TDM) offers a systematic framework for optimizing pharmacotherapy at the individual patient level, by tailoring drug dosing to ensure maximum therapeutic efficacy while minimizing the risk of toxicity [1-3]. This process relies on the precise quantification of drug concentrations in biological fluids, typically blood or plasma, where the timing of sampling is critical to accurately estimate drug exposure [1-3]. By interpreting timely sampled (traditionally at trough and steady state) drug concentrations in biological fluids within the context of established pharmacokinetic-pharmacodynamic relationships, clinicians can propose informed dosing adjustments that align with the patient’s specific pharmacokinetic profile and ensure optimal outcomes [1-3]. This strategy has been recommended for various drugs across oncology, infectious diseases, immunology, psychiatry, and other specialties, aiming to deliver at the right dose and time for each patient [1]. Despite these advantages, traditional TDM remains limited by its reliance on centralized laboratories, slow analytical turnaround, need to wait for steady state before sampling, simplistic “rule-of-three” dosing adaptations poorly adapted to drugs with nonlinear pharmacokinetics, and heterogeneous clinician awareness and guideline availability, all of which delay or hinder truly individualized dosage optimization [1,3]. The operational burden of this process is substantial, requiring extensive collaboration among diverse health care stakeholders [2,4]. The fragmentation of tasks across preanalytical, analytical, and postanalytical stages creates numerous vulnerabilities, rendering the entire workflow prone to systemic errors [2,4]. A recent review reported that preanalytical factors (including steps prior to sample preparation for analysis) account for the majority of laboratory errors, ranging from 46% to 68% of total testing errors, with approximately 26% of these failures exerting a detrimental effect on patient care [4]. These errors are predominantly driven by timing inaccuracies, where studies have shown that up to 60% of specimens may be collected at inappropriate sampling times for traditional TDM, and 22% to 31% are drawn before steady state, rendering the results invalid for interpretation [4]. Furthermore, the omission of essential data, such as the time of the last dose intake or the current dosage regimen, frequently compromises the ability to interpret results accurately, a challenge that persists even with the implementation of computerized provider order entry systems [4].

Integrating model-informed precision dosing (MIPD) with point-of-care (POC) diagnostics creates a powerful synergy for personalized drug therapy [5-7]. Leveraging population pharmacokinetic (popPK) models and Bayesian forecasting, this method provides the best prediction of individual exposure from available medical data and drug measurements [5]. Crucially, MIPD software surpasses the limitations of standard TDM by accurately handling unconventional sampling times, where traditional TDM is unfeasible [5]. While performance depends on the validity of the popPK models in the targeted population, MIPD facilitates flexible monitoring through optimized sampling strategies, allowing for dose adjustments independent of steady-state achievement [5]. When coupled with bedside POC, this flexibility is fully realized, enabling rapid, decentralized sampling that significantly shortens the decision-making loop and minimizes process-related errors [6,8]. Though current MIPD platforms already mitigate many limitations of classical TDM, their clinical impact remains constrained by suboptimal workflow integration and the lack of embedded safeguards against erroneous inputs or implausible measurements [2,4,5].

Adding rule-based validation, alerts, and workflow features on top of an MIPD engine yields a clinical decision support system (CDSS) that outperforms individualized dosing strategies [9-11]. By checking data plausibility, flagging potential reporting errors, standardizing the interpretation of exposure against therapeutic targets, and delivering concise recommendations within the routine workflow, a CDSS stands as the closest substitute for a TDM expert [9-11]. Although MIPD could, by definition, be regarded already as a form of CDSS, since it transforms patient data into dosing recommendations, the term CDSS is reserved here for the combination of an MIPD core with an integrated validation system delivering actionable recommendations [9-11]. Well-designed CDSS seamlessly integrated into electronic health records can reduce preventable medication errors, shorten the interval between measurement and decision, and manage alert thresholds to detect true problems without causing alert fatigue and loss of trust [10,11].

Imatinib, the first small-molecule targeted anticancer agent to demonstrate unprecedented efficacy against specific malignancies, is characterized by marked interindividual pharmacokinetic variability. As a result, it has been confirmed that precise dosing is required to maintain steady-state trough concentrations within well-established therapeutic windows: specifically, >1000 ng/mL for chronic myeloid leukemia and >1100 ng/mL for gastrointestinal stromal tumors, while avoiding concentrations above 2000 to 3000 ng/mL associated with toxicity [12-14]. Imatinib TDM-guided dosing is associated with significantly improved clinical outcomes compared to standard care, including higher rates of major molecular and cytogenetic responses at 12 months (63% in the TDM-guided group vs 37% in the control group) [13,15,16]. Furthermore, cost-effectiveness analyses demonstrate that TDM yields improved quality-adjusted life years and lower overall costs [13,17,18]. As a result, imatinib TDM practice for patients with chronic myeloid leukemia and gastrointestinal stromal tumor is formally endorsed by the IATDM-CT (International Association of Therapeutic Drug Monitoring and Clinical Toxicology) [19]. New targeted anticancer drugs tending to replace imatinib may also require TDM and dosage adjustment; however, there is still a lack of supporting evidence in this regard [13]. Furthermore, as these new drugs are more expensive than imatinib, now available in generic form, the latter remains recommended as first-line treatment, and its TDM is likely to delay drug rotation due to ineffectiveness or toxicity issues [20]. While imatinib TDM by POC measurement has been explored, its integration with MIPD, and particularly its embedding within a CDSS framework, remains novel, offering a unique opportunity for real-time, decentralized dose optimization in the outpatient setting.

This study aims to rigorously determine whether the proposed imatinib CDSS is suitable for subsequent clinical prospective evaluation by (1) quantifying its ability to detect data-quality issues and (2) characterizing its clinical performance in recommending effective and safe dosing adaptations. As such, it provides a proof of concept that underpins future real-life prospective trials and large-scale implementation.

## Methods

### popPK Model and Virtual Patients

We simulated virtual patients (N=60) using a validated structural one-compartment popPK model (Table 1), developed on real patients receiving imatinib followed at our institution, using MonolixSuite (version 2024R1; Simulations Plus) [21,22].

**Table 1.** Parameters of the imatinib population pharmacokinetic model.

Parameter	Value
CL^a^ (L/h)	11
*ω*^b^_Cl_ (CV%^c^)	33
*V*_d_^d^ (L)	365
*ω_V_*_d_ (CV%)	58
*k*_a_^e^ (h^–1^)	1.28
*σ*_prop_^f^ (CV%)	34

a CL: typical clearance.

b *ω*: interindividual variability parameter.

c CV: coefficient of variation.

d *V*_d_: typical central volume of distribution.

e *k*_a_: typical first-order absorption rate constant.

f *σ*_prop_: proportional residual error.

We used specific dosing regimens of 100 mg, 300 mg, 200 mg, 400 mg, 600 mg, or 800 mg once daily, and 200 mg, 400 mg, 600 mg, or 800 mg twice daily. To ensure clinically relevant pharmacokinetic profiles, individual pharmacokinetic parameters were sampled from their original distributions, corresponding to elimination half-lives between 3 and 48 hours, and were used to simulate *true* concentrations (*C*_true_) from treatment initiation up to steady state by day 11 of treatment. We added a proportional individual-dependent residual error (residual unexplained variability [RUV]) to simulate *observed* concentrations (*C*_obs_). We stratified the population into 3 equal groups of 20, based on steady-state *observed* trough concentrations (*C*_min,obs_), to ensure necessary intervention: underexposed (<500 ng/mL), within target (1100‐2000 ng/mL), and overexposed (>3000 ng/mL), excluding ambiguous cases close to therapeutic boundaries (500‐1100 ng/mL and 2000‐3000 ng/mL). To assess the robustness of TDM interventions, we imputed single data-quality issues reflecting clinical routine into 45 of the cases (Table 2 and Table S1 in Multimedia Appendix 1), leaving 15 as valid steady-state samples. These data-quality scenarios were categorized into 6 distinct groups (Table 2 Table S1 in Multimedia Appendix 1). The first 2 groups defined the scope of data considered valid for the TDM expert, for whom interpretability was strictly limited to samples drawn at the trough or within a window permitting acceptable extrapolation (up to 6 h before the steady-state trough). These included (1) valid data and (2) extrapolation valid for TDM expert and CDSS. In contrast, the CDSS demonstrated a broader scope of technical validity due to its MIPD-based architecture, enabling the processing of (3) extrapolation valid for CDSS (EC). This category comprised samples drawn during the absorption phase or explicitly declared as not at steady state. While technically processable by the CDSS, provided these conditions were correctly declared, they were classified as invalid for standard interpretation by the TDM expert. The remaining categories included the following: (4) blocking issues (BIs), involving critical data conflicts such as chronological timestamp inversion or date-format transposition that technically preclude any analysis; (5) extrapolation issues (EIs), where non–steady-state samples were incorrectly classified as steady-state; and (6) miscellaneous issues (MIs), a heterogeneous group encompassing treatment initiation date shift, sampling-time rounding, sample mislabeling, dosage transcription error, and concentrations below the lower limit of quantification set at 100 ng/mL. We compiled the 60 simulated cases in a Microsoft Excel spreadsheet, each one contributing a single *C*_min,obs_.

**Table 2.** Classification, description, and distribution of virtual patients and errors.

Category and coding	Short description	Number of patients (N=60)
Valid		
VTC	Valid steady-state trough sample	15
Extrapolation valid for TDM^a^ expert and CDSS^b^		
ETC	Pretrough sample requiring extrapolation (up to 6 h before trough)	3
Extrapolation valid for CDSS		
EC1	Absorption phase sample requiring extrapolation (1 h to 3 h postdose)	3
EC2	Non–steady-state sample (explicitly declared)	3
Blocking issue		
BI1	Chronological inversion of timestamps (initiation, dose, or sampling)	6
BI2	Date format transposition (day/month swap) in timestamps	3
Extrapolation issue		
EI	Non–steady-state sample (misclassified as steady-state)	3
Miscellaneous issue		
MI1	Shift in treatment initiation date (+6 d to 9 d)	3
MI2	Sampling time rounding discrepancy (15 min to 60 min before trough)	6
MI3	Absorption phase sample (1 h to 3 h postdose) mislabeled as trough	3
MI4	Trough sample mislabeled as absorption phase (1 h to 3 h postdose)	3
MI5	Dosage regimen transcription error	6
MI6	Concentration below the lower limit of quantification (virtually fixed at 100 ng/mL)	3

a TDM: therapeutic drug monitoring.

b CDSS: clinical decision support system.

### Ethical Considerations

This validation study was conducted entirely *in silico* using simulated virtual patient profiles generated from a popPK model. The underlying clinical data used to develop the popPK model were collected in accordance with institutional guidelines at Lausanne University Hospital and approved by the Ethics Committee of the Canton of Vaud (project number: 2024‐01723). Informed consent for research participation was systematically obtained from all patients prior to data extraction through the institution’s general consent framework.

### CDSS Development

We adopted as CDSS the software developed in the context of the TuberXpert project [23] under an open-source license (AGPLv3 [GNU Affero General Public License version 3]) as part of the sotalya ecosystem [24]. Though originally targeted to rifampicin, the software is very generic and, given a suitable drug popPK model (defined in an XML file), can be applied to a vast spectrum of drugs. The drug model file embeds all the mathematical information a popPK model offers to compute drug circulating concentrations: model type, typical pharmacokinetic parameter values along with their interindividual variability, influence of specific covariates on these parameters, and residual error model. It allows the computation of dosage adjustments, specifically targeting a *C*_min_ of 1500 ng/mL (within the established 1100‐2000 ng/mL therapeutic range) in the cases of this study, but also the automatic detection of potential anomalies in the sampling data. The computational core used by this CDSS system is Tucuxi [25], another open-source software [24] that specifically handles all numerical aspects of the modeling.

The CDSS software requires, as input, among other optional data, the dosage history; dosing and sampling times; and covariate information for each patient. We therefore parsed the spreadsheet prepared for the TDM expert with a Python script and generated the input files required by Tucuxi. A first set of checks was applied at this stage, discarding patients for whom either the concentration measurement date or the last dose date preceded the treatment start date, issues corresponding to data entry errors. We then fed the generated input files to the CDSS and automatically parsed the generated reports to either detect further errors or extract useful data: (1) the suggested dosing scheme; (2) the (optional) discontinuation interval; (3) any warnings due to either statistical inconsistencies with respect to the population model described in the drug file or inefficacy/toxicity alerts. In particular, the measurement result was checked to be within the 10th to 90th expected percentile range for each dosage regimen, and the dates were further controlled to ensure that the sample was meaningful (ie, not too far in time with respect to the last dose intake).

Internally, Tucuxi-CDSS is organized in three processing layers: (1) data validation and preprocessing, which performs consistency checks and flags preanalytical errors; (2) model-based computation, which queries the Tucuxi-Core engine to derive percentile-based plausibility assessments and Bayesian a posteriori parameter estimates; and (3) decision logic and reporting, which compares predicted exposure metrics to configurable therapeutic targets and assembles a structured clinical recommendation. The system is highly configurable through XML files that define not only the drug models but also covariate boundaries with soft and hard thresholds, language settings, and institution-specific reporting templates, enabling deployment across diverse clinical environments. A detailed description of the system architecture, operational workflow, and verification strategy is provided on readthedocs [26] and in reference [27].

### Resolution of the Virtual Cases by a TDM Expert or a CDSS

The same 60 virtual cases were independently assessed by both the CDSS and a TDM expert (PT), a pharmacist holding a PhD in clinical pharmacology and head of the TDM unit at the Service of Clinical Pharmacology of the University Hospital of Lausanne. While informed of the possible presence of data-quality errors, both evaluators remained blinded to the specific nature and frequency of these issues. Error detection was assessed by requiring evaluators to explicitly specify any suspected data issue, using methodology-specific tools and, notably, the 10th to 90th percentile popPK curves for each imatinib dosing regimen as a common reference range for both the expert and the CDSS. These curves represent the likelihood of having a concentration within this range given the variability in the population. Figure 1 schematically summarizes the study methodology.

**Figure 1.**
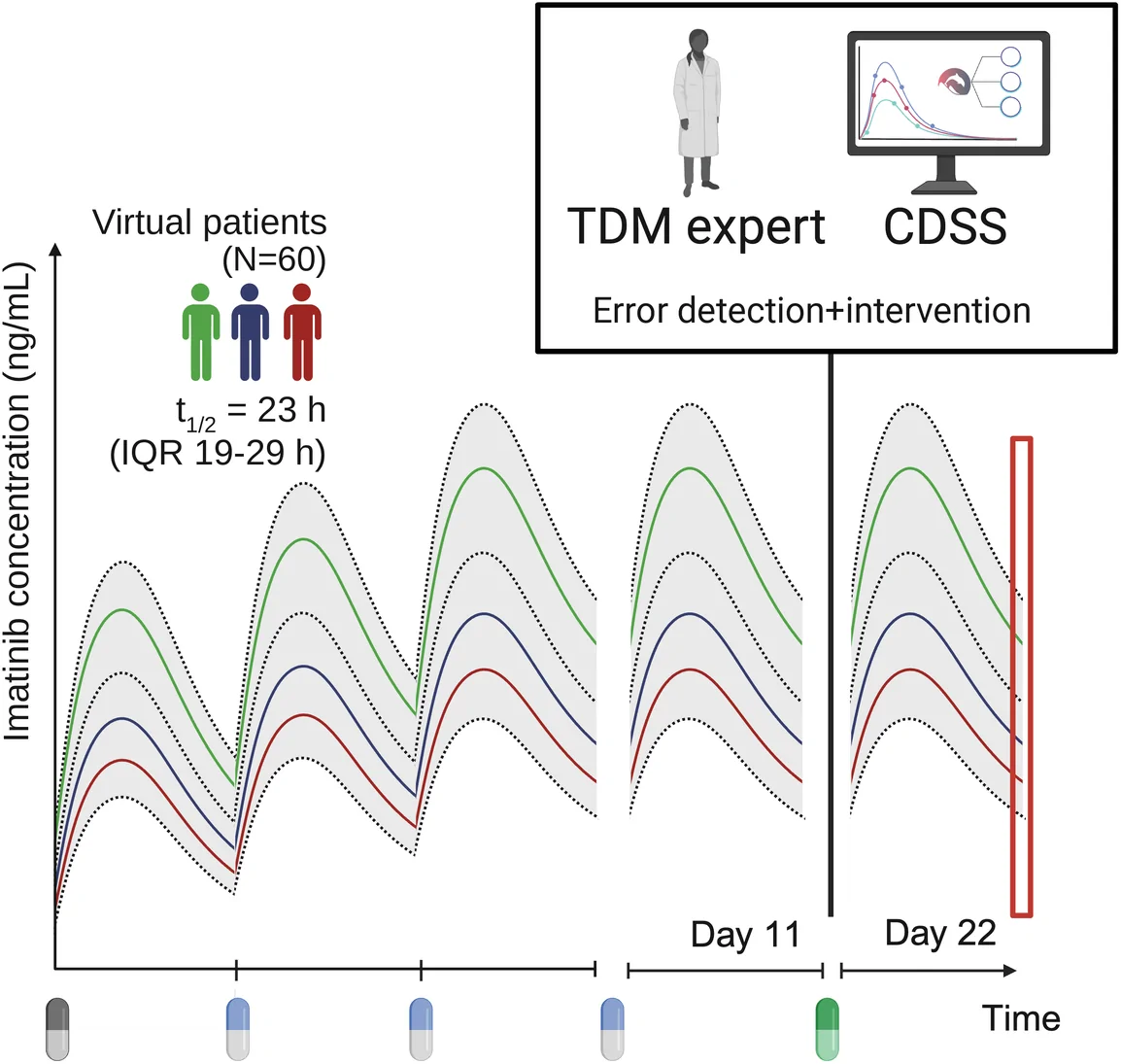
Schematic representation of the *in silico* study methodology. A virtual cohort (N=60) was simulated using a population pharmacokinetic (popPK) model, incorporating metabolic diversity with a median half-life (*t*_1/2_) of 23 (IQR 19‐29) hours. The cohort was stratified into 3 subsets: cases valid for both the clinical decision support system (CDSS) and the therapeutic drug monitoring (TDM) expert (n=18), cases valid only for the CDSS (n=6), and invalid cases (n=36). Following the initiation of imatinib therapy (gray pill), a primary TDM evaluation was performed on day 11 (steady state), where a head-to-head comparison was conducted between the human expert and the CDSS to assess error detection and formulate dosing adjustments (green pill). Subsequent simulations were performed based on these interventions to evaluate their impact on steady-state concentrations at day 22 (red box), where final target attainment was compared between both arms. The shaded area represents the population prediction interval derived from the underlying popPK model. The different curve colors (green, blue, and red) represent examples of patients’ imatinib pharmacokinetic profiles.

### Error-Detection Performance

To evaluate the accuracy of error detection of the TDM expert and the CDSS in identifying data-quality issues, sensitivity (equation 1), specificity (equation 2), precision (positive predictive value, equation 3), and the *F*_1_-score (harmonic mean of precision and sensitivity, equation 4) were calculated based on the classification of cases as either valid or erroneous [28]. Corresponding 95% CIs were computed using the Clopper-Pearson exact method for sensitivity, specificity, and precision, and statistical comparability was assessed by examining the overlap of these intervals [29]. These metrics were defined as follows:


(1) $$Sensitivity=\;\frac{TP}{TP+FN}$$

(2) $$Specificity=\;\frac{TN}{TN+FP}$$

(3) $$Precision=\;\frac{TP}{TP+FP}$$

(4) $$F_{1}=2\times \;\frac{Precision\;\times Sensitivity}{Precision+Sensitivity}$$

where TP represents true positives (correctly identified issues), TN represents true negatives (correctly identified valid cases), FP represents false positives (valid cases incorrectly flagged as issues), and FN represents false negatives (missed issues).

### Clinical Outcomes After TDM Expert or CDSS Intervention Analysis

Following this validation step, both evaluators aimed to achieve a steady-state trough target of 1100ng/mL to 2000 ng/mL by proposing dosing adjustments. Following their respective dosing interventions, the resulting true steady-state trough concentrations (*C*_min,true_), assumed to be reached 22 days after treatment initiation, were simulated using the individuals’ true pharmacokinetic parameters. In instances where no dosing adjustment or treatment discontinuation was proposed, the simulation assumed the continuation of the initial dosing regimen. Clinical outcomes were assessed at baseline (day 11 posttreatment initiation) and compared to the postintervention outcomes observed at day 22 posttreatment initiation. Cases were then classified into 6 mutually exclusive categories based on baseline and day 22 therapeutic target attainment (1100‐2000 ng/mL) [14]: “effective” when a patient off-target at baseline achieved the target following a dosing adjustment; “unnecessary” when a patient already within target received a dosing adjustment and remained within target; “critical” when an underexposed patient became overexposed following a dosing adjustment; “ineffective” when a patient remained underexposed or overexposed, or shifted from overexposure to underexposure after a dosing adjustment; “detrimental” when a patient initially within target fell outside the target at day 22 regardless of whether a dosing adjustment had been made (ie, patients who moved outside the target because of pharmacological drift despite no adjustment belong to this category); and “no intervention” when no dosing adjustment was proposed and the patient did not meet the definition of the “detrimental” category. Statistical discordances between the TDM expert and CDSS interventions were evaluated using the McNemar test [30].

### Subset Analyses

To accurately evaluate the system’s ability to flag data-quality issues, error-detection performance was assessed on 2 distinct sets. The primary analysis was conducted on the full set (N=60), encompassing all simulated scenarios. A secondary, stratified analysis was performed on a restricted subset (n=54), specifically excluding the 6 EC cases. This exclusion was necessary to neutralize the validation disparity between the 2 methods, as these specific cases represented samples that were correctly identified as valid by the CDSS logic but were inherently considered erroneous or unusable by the TDM expert due to the limitation introduced previously.

Further detailed assessments of clinical interventions were performed across 3 subpopulations to isolate different performance drivers. Beyond the full dataset (N=60), an initial exclusion set (exclusion 1, n=51) was established by removing all BI (BI1 and BI2), which represented timestamp errors that technically precluded any dosing intervention by either method. To facilitate a rigorous head-to-head comparison, a second, refined set (exclusion 2, n=39) was created by further excluding cases that introduced methodological asymmetry. This included the removal of ECs, as well as EIs and mislabeled absorption-phase samples (MI3), thereby isolating a subset of clinical scenarios in which both the expert and the CDSS had a theoretically equal opportunity to propose valid dosing adjustments.

## Results

### Error Detection

Overall, the TDM expert and the CDSS demonstrated comparable error-detection capabilities, with widely overlapping CIs suggesting no substantial difference in performance (Table 3 and Table S2 in Multimedia Appendix 1). In the standard set (N=60), the human TDM expert demonstrated perfect specificity (100%, 95% CI 81%‐100%) and precision (100%, 95% CI 82%‐100%), detecting no false positives. However, the expert achieved a lower sensitivity (45%, 95% CI 30%‐61%) compared to the CDSS (53%, 95% CI 35%‐70%; Table 3). Consequently, the CDSS exhibited a slightly higher *F*_1_-score (64% vs 62%), despite a lower specificity (83%, 95% CI 63%‐95%), resulting from a higher false-positive rate than that of the TDM expert. When excluding the 6 ECs (n=54), the expert’s sensitivity improved to 50% (95% CI 33%‐67%), while the CDSS’s specificity increased to 89% (95% CI 65%‐99%). In this refined analysis, both methods achieved an identical *F*_1_-score of 67% (Table S2 and Figure S1 in Multimedia Appendix 1). Figure S2 in Multimedia Appendix 1 offers a detailed count of the true positive and false negative rates stratified by error types.

**Table 3.** Comparative analysis of error-detection performances (N=60).

Metric	TDM^a^ expert, % (95% CI)	CDSS^b^, % (95% CI)
Sensitivity	45 (30‐61)	53 (35‐70)
Specificity	100 (81‐100)	83 (63‐95)
Precision	100 (82‐100)	83 (61‐95)
*F*_1_-score^c^	62	64

a TDM: therapeutic drug monitoring.

b CDSS: clinical decision support system.

c As the *F*_1_-score is a composite metric that does not follow a binomial distribution, no exact Clopper-Pearson CI is available, and it is reported as a point estimate only.

### Clinical Outcomes After TDM Intervention

In the full standard set (N=60), both the TDM expert and the CDSS significantly improved the proportion of patients reaching the therapeutic target compared to baseline, from 22% (n=13) to 37% (n=22, *P*=.02) and 55% (n=33; *P*<.001), respectively (Figures 2 and 3, Table 4, and Table S4 in Multimedia Appendix 1). The CDSS achieved a statistically significantly higher proportion of target attainment compared to the expert (*P*=.046). In the exclusion 1 set excluding BIs (n=51), the CDSS maintained a higher success rate (n=32, 63%) compared to the expert (n=19, 37%; *P*=.01; Table S4 and Figure S3 in Multimedia Appendix 1). In the exclusion 2 set (n=39) designed to most fairly compare head-to-head TDM experts and the CDSS, the latter still outperformed the TDM expert, with 69% (n=27) of patients reaching the target vs 41% (n=16) for the expert (*P*=.01; Table S4 and Figure S3 in Multimedia Appendix 1).

**Figure 2.**
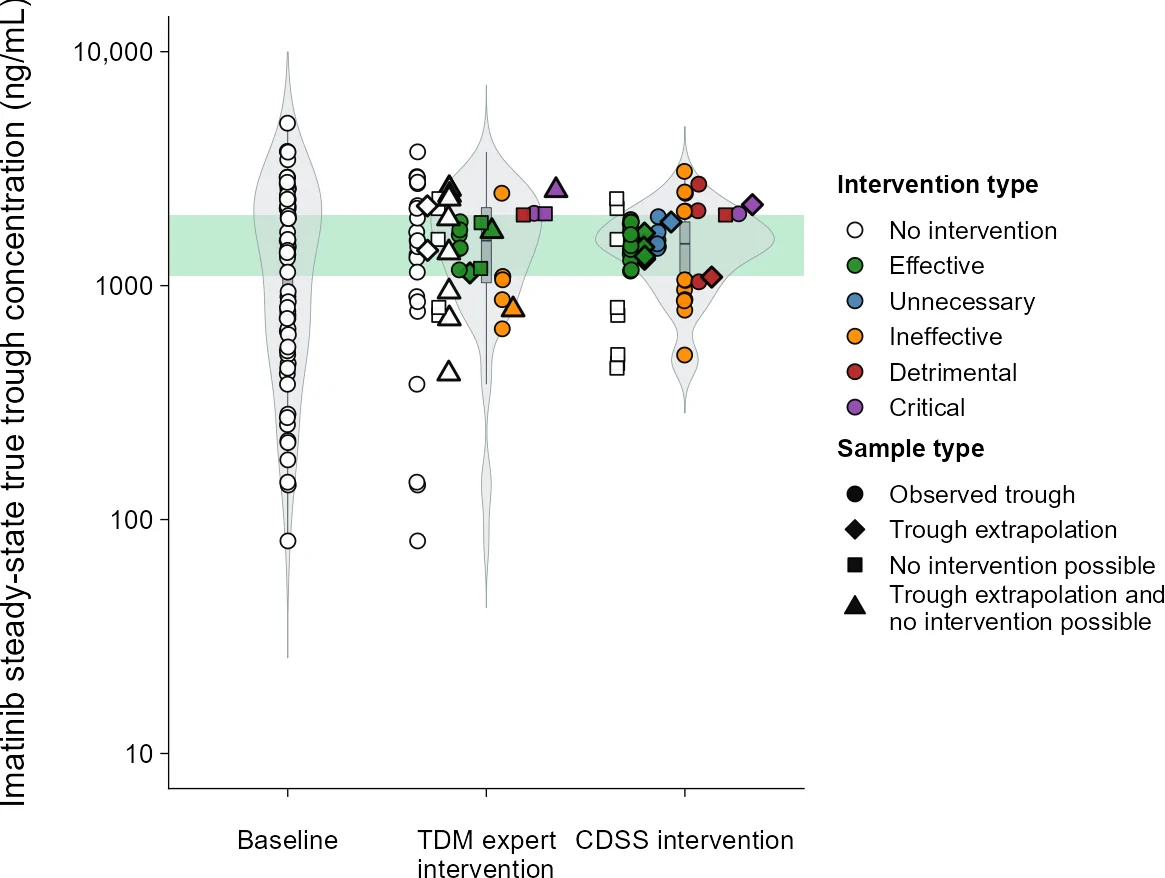
Distribution of steady-state true trough concentrations of imatinib at baseline and after therapeutic drug monitoring (TDM) expert or clinical decision support system (CDSS) intervention (N=60). The green band depicts the therapeutic target (1100‐2000 ng/mL) for the steady-state trough concentration [14]. Colors indicate the intervention outcome: green indicates effective interventions where patients initially underexposed or overexposed attained the target; blue represents unnecessary interventions for patients within target at baseline who received a dosing adjustment and remained within target; orange denotes ineffective interventions where patients remained underexposed or overexposed or shifted from overexposure to underexposure despite dosing adjustment; purple represents critical intervention where patients were underexposed but turned overexposed after dosing adjustment; red signifies detrimental interventions where patients initially within target fell outside the target regardless of dosing adjustment; and white stipulates no intervention (absence of dosing adjustment, unless classified as detrimental). Shapes represent the sample type: circles indicate observed troughs at steady state; diamonds concentrations requiring extrapolation to the trough; squares cases where no intervention was possible; and triangles cases requiring extrapolation precluding intervention.

Analysis of intervention types revealed 5 detrimental cases in the CDSS (Table 5, Tables S5 and S3 in Multimedia Appendix 1, Figures 2 and 4, and Figures S4 and S5 in Multimedia Appendix 1): 1 patient each in the EC1, EI (misclassified steady-state), MI2 (sampling time rounding), and MI4 (sample mislabeling) categories, and a specific pharmacological deviation was observed in a BI case (BI2) common to the TDM expert intervention (Table 5 and Table S5 and Figure S5 in Multimedia Appendix 1).

**Figure 3.**
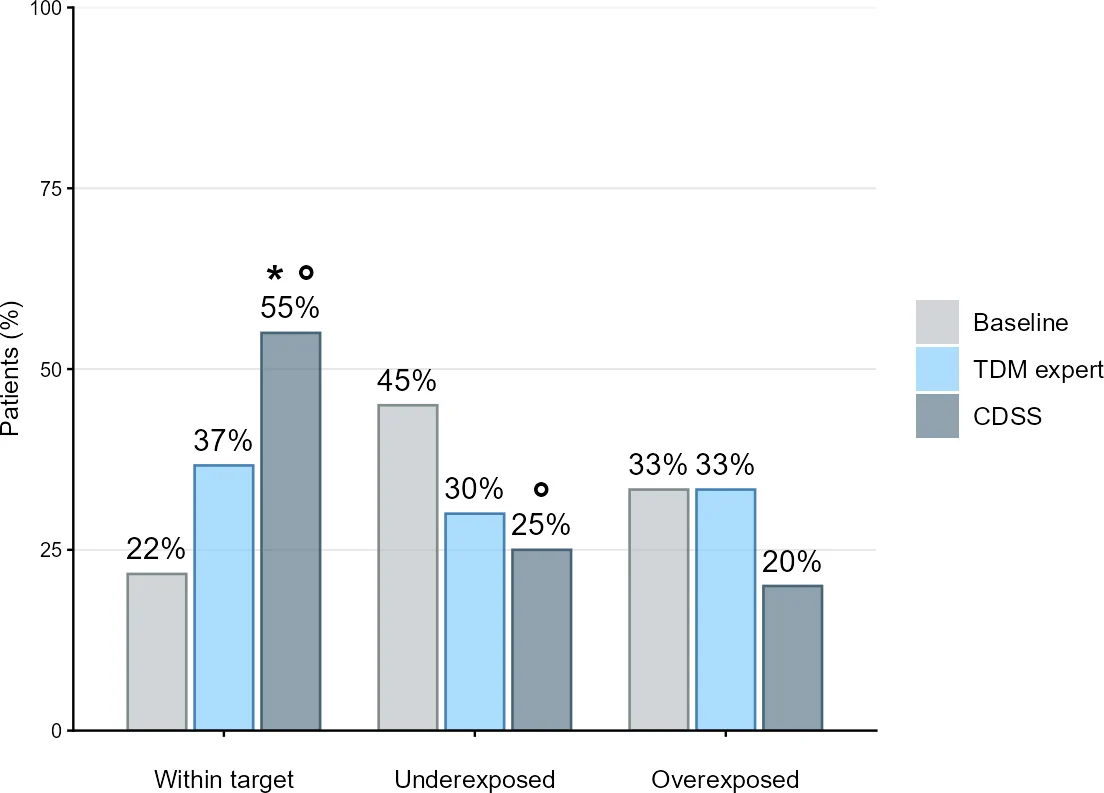
Distribution of patients across clinical outcomes after therapeutic drug monitoring (TDM) expert or clinical decision support system (CDSS) intervention (N=60). The asterisk (*) and empty circle (°) indicate statistical significance (*P*<.05) of the difference between CDSS and either TDM expert or baseline, respectively [30].

**Table 4.** Comparative analysis of clinical outcomes after the intervention (N=60).

Clinical outcome	Baseline, n (%)	TDM^a^ expert intervention, n (%)	CDSS^b^ intervention, n (%)	*P* value (expert vs baseline)	*P* value (CDSS vs baseline)	*P* value (CDSS vs expert)
Within target	13 (22)	22 (37)	33 (55)	.02	<.001	.046
Underexposed	27 (45)	18 (30)	15 (25)	.02	.01	.61
Overexposed	20 (33)	20 (33)	12 (20)	>.99	.10	.06

a TDM: therapeutic drug monitoring.

b CDSS: clinical decision support system.

Conversely, the CDSS demonstrated a statistically significantly higher rate of “effective” interventions (25 cases vs 10 cases; *P*=.002), whereas the expert more frequently proposed “no intervention” strategies (39 cases vs 8 cases; *P*<.001; Figures 2 and 4, Table 5, Tables S3 and S5 in Multimedia Appendix 1). As shown in Figure 2 and Table 5 and Tables S3 and S5 in Multimedia Appendix 1, the CDSS significantly proposed unnecessary dose adjustments in 7 cases, whereas the TDM expert correctly avoided these entirely (*P*=.02). The frequency of ineffective interventions was statistically comparable between the 2 methods, with 7 instances for the expert vs 13 for the CDSS (*P*=.11), and similar results were obtained when exclusions were applied.

**Table 5.** Comparative analysis of outcomes by intervention category (N=60).

Category	TDM^a^ expert (n)	CDSS^b^ (n)	*P* value
No intervention	39	8	<.001
Effective	10	25	.002
Unnecessary	0	7	.02
Ineffective	7	13	.11
Detrimental	1	5	.13
Critical	3	2	>.99

a TDM: therapeutic drug monitoring.

b CDSS: clinical decision support system.

**Figure 4.**
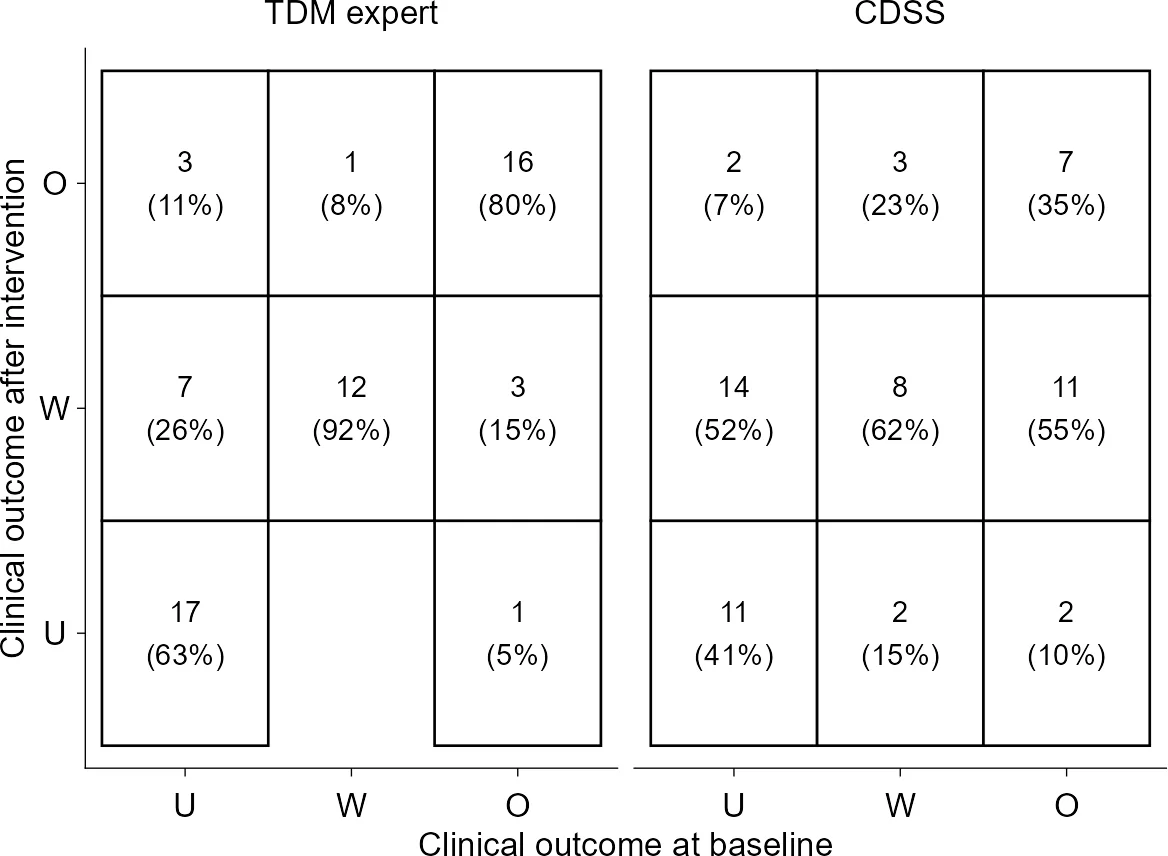
Representation of clinical outcomes after intervention of clinical decision support system (CDSS) or therapeutic drug monitoring (TDM) expert (N=60). Within therapeutic target (W) was defined as 1100 ng/mL to 2000 ng/mL for a trough steady-state concentration, and underexposed (U) and overexposed (O) were defined as below and above these thresholds, respectively [14].

## Discussion

### Principal Findings

This *in silico* study compared the performance of a rule-based CDSS and a human TDM-expert in optimizing therapeutic target attainment using imatinib as a case study and showed enhanced performance of the CDSS over the TDM expert. The CDSS consistently outperformed the expert across a variety of settings, representative of “real-life” scenarios, allowing a refined head-to-head comparison designed to ensure that both the software and the expert could actively intervene. Regarding safety, although the global reduction of off-target exposure suggests a net benefit, it is important to note that 5 patients who were initially within the therapeutic window drifted off-target following CDSS recommendations. This highlights that, while the algorithm effectively reduces the total number of patients outside the therapeutic window compared to the expert, its safety remains fairly contingent on data integrity.

This work provides several novel contributions to the field of digital precision medicine. Specifically, it offers the first validation of a “nonexpert” CDSS architecture integrating MIPD with a rule-based validation layer designed for decentralized, POC settings specifically for imatinib. Furthermore, it establishes a necessary technical benchmark by providing the first head-to-head performance evaluation between such an automated system and a human TDM expert, specifically focusing on system resilience by simulating a “chaotic” environment with a 75% error prevalence, allowing for a rigorous assessment of the CDSS as a digital gatekeeper for data integrity. Ultimately, by demonstrating that an automated system can achieve comparable or superior performance to an expert baseline, this study provides the essential proof-of-concept required to transition toward prospective, real-life clinical trials of automated precision dosing.

A key driver of differences in target attainment between the CDSS and the TDM expert was the contrasting intervention strategies. As clinical outcomes were evaluated based on the steady-state *C*_min,true_, the high RUV (34%) makes the estimation of this true exposure from a single observed concentration, *C*_min,obs_ difficult for a human expert without computational aid. In contrast, the CDSS uses Bayesian forecasting to estimate the steady-state trough, effectively “seeing through” the measurement noise. In addition, the TDM expert frequently adopted a more conservative approach, choosing “no intervention” when facing slightly off-target exposure, while the study relied on a rigid therapeutic threshold (1100‐2000 ng/mL). In clinical reality, experts do not infrequently incorporate a wider “margin of tolerability,” accepting marginally out-of-range concentrations to avoid disrupting a stable patient by iterative dosing adaptations. By strictly penalizing deviations outside this narrow window, our evaluation metric may inherently favor the CDSS’s mathematical precision over the expert’s pragmatic, clinically anchored approach. Consequently, the dosing adjustments proposed by the CDSS globally bring patients closer to the targeted steady-state trough of 1500 ng/mL, despite rare instances of detrimental adjustments. While the CDSS’s active optimization strategy appears able to drive higher efficacy, its translation into routine practice must account for clinical inertia, where clinicians may be appropriately reluctant to change established regimens, as previously reported [31]. Additionally, the expert’s active dosing adjustments in 2 chronological inversion cases (BI1) likely reflect either routine clinical pragmatism or an overlooked documentation error during manual review, contrasting with the CDSS’s strict, rule-based execution blocks.

The relatively low sensitivity observed for both the TDM expert (45%, 95% CI 30%-61%) and the CDSS (53%, 95% CI 35%-70%) highlights a critical dependency on the statistical thresholds used for error detection. Both methods rely on popPK percentile curves to assess the biological plausibility of a sample (computed for the CDSS and plotted for the TDM expert); in this study, the 10th and 90th percentiles were used as boundaries for expected concentrations. While this broad interval maximizes specificity (100%, 95% CI 81%-100% and 83%, 95% CI 63%-95% for the TDM expert and the CDSS, respectively) by reducing false alarms, it inherently limits the ability to flag subtler deviations, such as certain EIs or MIs that may still fall within those wide confidence bands. There is a direct, adjustable trade-off between sensitivity and specificity governed by these thresholds. Adopting more stringent boundaries, for instance, narrowing the acceptance range to the 20th and 80th percentiles, would increase the sensitivity, ensuring that fewer errors go undetected. Given that the CDSS should prioritize safety, maximizing sensitivity is often the priority to “rule out” potential data-quality issues [28]. However, this shift would come at the cost of reduced specificity, potentially generating more false positives and increasing the risk of “alert fatigue” for clinicians, and further hindering proper adherence toward dosing recommendations [10,32]. The calibration of error-detection sensitivity necessitates a critical balance between clinical safety and therapeutic effectiveness. On one hand, adopting more stringent detection boundaries enhances safety by effectively flagging errors, thereby preventing the CDSS from proposing harmful, detrimental dosing adaptations based on flawed data and predictions. On the other hand, such hyper-vigilance may disproportionately penalize “physiologically extreme” but valid patients whose pharmacokinetic profiles naturally reside within the tails of the population distribution. By incorrectly categorizing these biological outliers as data errors, the system may lead to a conservative “no intervention” approach from the end-user, thus withholding necessary dose optimizations and leaving the patient outside the therapeutic target. Consequently, maximizing the clinical utility of the tool requires an optimized threshold that serves as a rigorous digital gatekeeper against noise without compromising the active optimization required to accommodate inherent biological diversity. Future implementations of the CDSS should therefore explore adjustable sensitivity settings, allowing institutions to calibrate this balance according to their specific tolerance for risk versus workflow interruption. Finding the optimal balance will require formal usability studies involving end-users to determine the threshold where the safety benefit of error detection remains acceptable relative to the clinical burden of alert fatigue.

Regarding the 4 of the 5 detrimental cases that arose from active interventions observed exclusively with the CDSS, the limited sample size stratified by error type precludes the identification of a systematic failure mode. These cases were not concentrated within a specific error category, but were distributed singly across the EC1, EI, MI2, and MI4 subgroups. Furthermore, in each instance, the remaining replicates within the same error category (2 or 5 cases) were not detrimental. This suggests that these isolated failures are likely attributable to probabilistic factors inherent to individual concentrations predicted by the CDSS, rather than a structural inability of the CDSS to handle specific error types, although the scarcity of data precludes definitive conclusions. Overall, further studies using substantially larger datasets are required to determine whether specific patterns underlie these detrimental outcomes. At this stage, the error-detection logic relies on a relatively rudimentary approach using population percentile curves to flag biological implausibility. While this method is precisely the one currently used in clinical routine, it lacks the resolution to consistently distinguish measurement noise from true physiological shifts when relying on a single data point. Consequently, the development of a dedicated module specifically engineered for error detection, beyond simple percentile boundaries, will be a primary focus for future iterations of the CDSS to further mitigate these safety risks. In addition, a single instance of a detrimental outcome was identified in a BI case (BI2), where the nature of the error technically precluded any dosing adaptation by either the expert or the CDSS. In this scenario, the patient drifted out of the therapeutic target independently of any intervention. This deviation highlights a discrepancy between clinical conventions and pharmacokinetic reality regarding the definition of steady-state. While clinical practice typically assumes steady state to be reached at 97% after 5 half-lives, slight accumulation continues beyond this point. In this specific case, the patient’s *C*_min,true_ was borderline at the upper therapeutic limit after 11 days of treatment. Consequently, the slight additional accumulation that occurred after the 22 days of treatment was sufficient to push the concentration above the threshold. Thus, this artifact underscores the limitations of enforcing stringent, binary therapeutic target limits without accounting for the natural pharmacological drift inherent to borderline cases. Moreover, in the real world, biological fluctuations further complicate the picture.

### Clinical Implications

By demonstrating that a CDSS can match or exceed expert performance in detecting errors and optimizing doses, we provide a pathway for decentralizing expert-level TDM. Currently, high-quality TDM is often restricted to specialized centers with on-site TDM experts. A reliable CDSS acts as a supportive device, allowing nonspecialized clinicians in smaller clinics or resource-limited settings to provide the same level of precision dosing as a dedicated expert while ensuring safe use by enabling the detection of errors. Furthermore, the system’s resilience to data-entry errors suggests it can serve as a critical safety buffer in high-pressure clinical environments where human cognitive fatigue may occur.

### Limitations

Several limitations of this study must be acknowledged. First, the investigation was purely *in silico*, which inherently simplifies the complexities of clinical practice. The popPK model used is intrinsically a coarse representation of reality. Furthermore, evaluating cases via a structured spreadsheet may not fully replicate the cognitive environment of routine TDM practice. While this format minimizes clinical “noise” by providing only the strict minimum data required for TDM (eg, dosing history, sampling time, and concentration), it lacks the visual safety guardrails and patient history available in modern electronic health records systems. The repetitive nature of assessing 60 complex cases in this manner may have introduced a further cognitive load or fatigue not typically present during isolated clinical interventions, potentially impacting the expert’s detection performance. Moreover, the investigation used the same popPK model for both virtual patient generation and CDSS evaluation. This form of circular modeling bias has nevertheless the merit of evaluating purely the intrinsic potential for dosage adjustment, while getting rid of model validity issues. While the substantial RUV (34%) mimics real-world measurement noise, true external validity would require evaluation against independent datasets. Second, unlike prospective real-life scenarios, the evaluators did not have access to patient history or prior dosing patterns, and the simulation assumed a single isolated data-quality error per individual. Consequently, the system was not fully tested against the chaotic data integrity issues not infrequently encountered in routine clinical practice, where compounded vulnerabilities, such as the simultaneous occurrence of timing inaccuracies and transcription errors, are plausible. Confronting the system with such overlapping errors could lead to unpredictable performance, where errors might either mask one another or, conversely, create extreme statistical outliers that are more easily detected. While the current study provides a necessary baseline characterization of system sensitivity to specific error types, future research must incorporate multifaceted error scenarios to better reflect real-world clinical complexity. Third, the study design introduced a potential bias by assuming automatic implementation of the CDSS’s recommendations. In a clinical setting, a physician would act as a gatekeeper: by bypassing this validation step, we could not assess “recommendation adherence” or the safety impact of a clinician potentially overriding the software. Fourth, statistical power was constrained by the sample size, particularly when stratified by specific error subtypes. With some error categories containing as few as 3 to 6 cases, robust statistical comparisons regarding the detection of specific error types or their associated outcomes were limited by low statistical power, increasing the risk of type II errors. Furthermore, the set was intentionally imbalanced to “stress-test” error detection, resulting in a high prevalence of errors or extrapolation (75%), however not much higher than some real-life estimations [4]. This left a small control group of valid cases for both the CDSS and the TDM expert (n=18), rendering the specificity estimate particularly imprecise: in such a limited sample, even a minimal number of false positives can disproportionately lower the calculated specificity. Fifth, informing the human evaluator beforehand about the potential presence of data-quality errors likely introduced a Hawthorne effect [33]. By priming the expert to be hyper-vigilant, the study may have measured an artificially heightened state of alert rather than a standard clinical cognitive baseline. However, this design choice probably induces a conservative bias; comparing the CDSS against a hyper-vigilant expert baseline demonstrates that the system’s detection capabilities remain comparable even to a clinician performing at peak alertness. In routine practice, where cognitive fatigue and lack of priming might lead to lower human vigilance, the relative advantage of an automated gatekeeper would likely be even more pronounced. Finally, the comparison relied on a single TDM expert, whose decision-making may not represent a generalized consensus; further trials involving more TDM experts will be necessary to implement the tool into clinical practice. However, while certain extrapolation scenarios inherently favored the CDSS’s mathematical flexibility over the expert’s conventional “rule of 3” constraints, it is notable that the CDSS retained its superior performance even in the restricted exclusion 2 subset where these technical biases were minimized.

### Conclusions

This *in silico* study validates the proposed CDSS as a robust tool for precision dosing, demonstrating error-detection performances globally comparable to those of a human TDM expert, while significantly outperforming the expert in therapeutic target attainment. While the CDSS’s active optimization strategy yielded superior overall efficacy, resulting in fewer patients outside the therapeutic window, safety remains a concern as detrimental interventions can sometimes be observed with the CDSS. Despite limitations inherent to the simulation design, these findings establish solid proof-of-concept for CDSS TDM. Consequently, this work lays the necessary groundwork for future prospective clinical trials, which remain a prerequisite for the routine implementation of such a CDSS.

## Supplementary material

10.2196/93304 Multimedia Appendix 1 Supplementary tables and figures presenting the classification and distribution of virtual patients, errors, and exclusion scenarios; comparative analyses of error-detection performance; stratified analyses of clinical outcomes and intervention results; and error-detection metrics and clinical outcomes following therapeutic drug monitoring expert or clinical decision support system intervention.

## Abbreviations

AGPLv3: GNU Affero General Public License version 3
BI: blocking issue
CDSS: clinical decision support system
EC: extrapolation valid for CDSS
EI: extrapolation issue
IATDM-CT: International Association of Therapeutic Drug Monitoring and Clinical Toxicology
MI: miscellaneous issue
MIPD: model-informed precision dosing
POC: point-of-care
popPK: population pharmacokinetic
RUV: residual unexplained variability
TDM: therapeutic drug monitoring
