# Supplementary material for: Safety and Performance Evaluation of a Clinical Decision Support System for Imatinib Therapeutic Drug Monitoring: In Silico Study
Source: JMIR Med Inform. 2026 Oct 21;14:e93304. doi: 10.2196/93304 (PMC13686438; doi:10.2196/93304)
Supplement: Supplementary file 1 [file medinform-v14-e93304-s001.docx]

# Supplementary Material: Tables

**Table S1**: Classification, description, and distribution of virtual patients, errors, and exclusion scenarios

| **Category** | **Coding** | **Short Description** | **Number of patients (n=60)** | **Prior steady-state sample** | **Prior trough sample** | **Validity of the data for TDM Expert** | **Validity of the data for CDSS** | **EC Exclusion** | **Ex.1** | **Ex.2** |
| --- | --- | --- | --- | --- | --- | --- | --- | --- | --- | --- |
| Valid | VTC | Valid steady-state trough sample | 15 | - | - | Yes | Yes | - | - | - |
| Extrapolation Valid for TDM Expert and CDSS | ETC | Pre-trough sample requiring extrapolation (up to 6h prior to trough) | 3 | - | Yes | Yes | Yes | - | - | - |
| Extrapolation Valid for CDSS | EC1 | Absorption phase sample requiring extrapolation (1–3h post-dose) | 3 | - | Yes | - | Yes | Yes | - | Yes |
|  | EC2 | Non-steady-state sample (explicitly declared) | 3 | Yes | Yes | - | Yes | Yes | - | Yes |
| Blocking Issue | BI1 | Chronological inversion of timestamps (initiation, dose, or sampling) | 6 | - | - | - | - | - | Yes | Yes |
|  | BI2 | Date format transposition (day/month swap) in timestamps | 3 | - | - | - | - | - | Yes | Yes |
| Extrapolation Issue | EI | Non-steady-state sample (misclassified as steady-state) | 3 | Yes | Yes | - | - | - | - | Yes |
| Miscellaneous Issue | MI1 | Shift in treatment initiation date (+6–9 days) | 3 | - | - | - | - | - | - | - |
|  | MI2 | Sampling time rounding discrepancy (15–60 min prior to trough) | 6 | - | Yes | - | - | - | - | - |
|  | MI3 | Absorption phase sample (1–3h post-dose) mislabelled as trough | 3 | - | Yes | - | - | - | - | Yes |
|  | MI4 | Trough sample mislabelled as absorption phase (1–3h post-dose) | 3 | - | - | - | - | - | - | - |
|  | MI5 | Dosage regimen transcription error | 6 | - | - | - | - | - | - | - |
|  | MI6 | Concentration below the lower limit of quantification (virtually fixed at 100 ng/mL) | 3 | - | - | - | - | - | - | - |

**Prior steady-state sample**: provided observed concentration is prior to the steady state; **Prior trough sample**: provided observed concentration is prior to the trough; **EC exclusion**: exclusion of extrapolation cases only valid for the CDSS; **Ex.1**: exclusion of the BI invalid for the CDSS; **Ex.2**: exclusion of the EC, BI, EI and **MI3** invalid either for the CDSS or the TDM expert.

**Table S2**: Exhaustive comparative analysis of error detection performances stratified by exclusion scenario

|  | Standard (n=60) | | EC exclusion (n=54) | |
| --- | --- | --- | --- | --- |
| **Metric** | **TDM expert (%) (CI_95%_)** | **CDSS (%) (CI_95%_)** | **TDM expert (%) (CI_95%_)** | **CDSS (%) (CI_95%_)** |
| Sensitivity | 45 (30-61) | 53 (35-70) | 50 (33-67) | 53 (35-70) |
| Specificity | 100 (81-100) | 83 (63-95) | 100 (81-100) | 89 (65-99) |
| Precision | 100 (82-100) | 83 (61-95) | 100 (81-100) | 90 (70-99) |
| F1 | 62 | 64 | 67 | 67 |

**Table S3**: Detailed count of patients stratified by clinical outcomes, intervention type, and error classification

|  | Baseline | | | | TDM Expert | | | | | | CDSS | | | | | |
| --- | --- | --- | --- | --- | --- | --- | --- | --- | --- | --- | --- | --- | --- | --- | --- | --- |
| **Coding** | **Total Number of Patients** | **Underexposed** | **Within Target** | **Overexposed** | **No Intervention** | **Effective** | **Unnecessary** | **Ineffective** | **Detrimental** | **Critical** | **No intervention** | **Effective** | **Unnecessary** | **Ineffective** | **Detrimental** | **Critical** |
| VTC | 15 | 3 | 4 | 8 | 9 | 4 | 0 | 1 | 0 | 1 | 0 | 9 | 4 | 2 | 0 | 0 |
| ETC | 3 | 1 | 1 | 1 | 2 | 1 | 0 | 0 | 0 | 0 | 0 | 2 | 1 | 0 | 0 | 0 |
| EC1 | 3 | 1 | 1 | 1 | 3 | 0 | 0 | 0 | 0 | 0 | 0 | 2 | 0 | 0 | 1 | 0 |
| EC2 | 3 | 2 | 0 | 1 | 2 | 0 | 0 | 0 | 0 | 1 | 0 | 2 | 0 | 0 | 0 | 1 |
| BI1 | 6 | 4 | 1 | 1 | 4 | 1 | 0 | 0 | 0 | 1 | 6 | 0 | 0 | 0 | 0 | 0 |
| BI2 | 3 | 1 | 1 | 1 | 1 | 1 | 0 | 0 | 1 | 0 | 2 | 0 | 0 | 0 | 1 | 0 |
| EI | 3 | 1 | 1 | 1 | 2 | 1 | 0 | 0 | 0 | 0 | 0 | 1 | 0 | 0 | 1 | 1 |
| MI1 | 3 | 1 | 1 | 1 | 1 | 1 | 0 | 1 | 0 | 0 | 0 | 1 | 1 | 1 | 0 | 0 |
| MI2 | 6 | 3 | 2 | 1 | 3 | 1 | 0 | 2 | 0 | 0 | 0 | 3 | 1 | 1 | 1 | 0 |
| MI3 | 3 | 2 | 0 | 1 | 2 | 0 | 0 | 1 | 0 | 0 | 0 | 0 | 0 | 3 | 0 | 0 |
| MI4 | 3 | 1 | 1 | 1 | 3 | 0 | 0 | 0 | 0 | 0 | 0 | 1 | 0 | 1 | 1 | 0 |
| MI5 | 6 | 4 | 0 | 2 | 4 | 0 | 0 | 2 | 0 | 0 | 0 | 2 | 0 | 4 | 0 | 0 |
| MI6 | 3 | 3 | 0 | 0 | 3 | 0 | 0 | 0 | 0 | 0 | 0 | 2 | 0 | 1 | 0 | 0 |
| Overall | 60 | 27 | 13 | 20 | 39 | 10 | 0 | 7 | 1 | 3 | 8 | 25 | 7 | 13 | 5 | 2 |

**VTC**: Valid data for the TDM expert and the CDSS; **ETC**: Extrapolation Valid for TDM Expert and CDSS; **EC**: Extrapolation Valid for CDSS; **BI**: Blocking Issue; **EI**: Extrapolation Issue; **MI**: Miscellaneous Issue. In two BI1 cases, the TDM expert nonetheless proposed a dosing adjustment despite the formal blocking issue; these are retained under their actual outcome (effective, critical) rather than reclassified as "no intervention", to reflect the evaluator's real response to the case.

**Table S4**: Stratified analysis of clinical outcomes following TDM expert or CDSS intervention

| Clinical Outcome | **Population Analysis** | **Patient Exclusion** | **N** | **Baseline**  **n(%)** | **TDM Expert Intervention**  **n(%)** | **CDSS Intervention**  **n(%)** | ***P*-value (Expert vs Baseline)** | ***P*-value (CDSS vs Baseline)** | ***P*-value (CDSS vs Expert)** |
| --- | --- | --- | --- | --- | --- | --- | --- | --- | --- |
| Within  Target | All patients | None | 60 | 13 (22) | 22 (37) | 33 (55) | .02 | <.001 | .046 |
|  | Ex.1 | BI1, BI2 | 51 | 11 (22) | 19 (37) | 32 (63) | .01 | <.001 | .01 |
|  | Ex.2 | EC1, EC2, BI1, BI2, EI, MI3 | 39 | 9 (23) | 16 (41) | 27 (69) | .02 | <.001 | .01 |
| Underexposed | All patients | None | 60 | 27 (45) | 18 (30) | 15 (25) | .02 | .01 | .61 |
|  | Ex.1 | BI1, BI2 | 51 | 22 (43) | 16 (31) | 10 (20) | .08 | .01 | .15 |
|  | Ex.2 | EC1, EC2, BI1, BI2, EI, MI3 | 39 | 16 (41) | 12 (31) | 6 (15) | .22 | .02 | .08 |
| Overexposed | All patients | None | 60 | 20 (33) | 20 (33) | 12 (20) | >.99 | .10 | .06 |
|  | Ex.1 | BI1, BI2 | 51 | 18 (35) | 16 (31) | 9 (18) | .68 | .052 | .10 |
|  | Ex.2 | EC1, EC2, BI1, BI2, EI, MI3 | 39 | 14 (36) | 11 (28) | 6 (15) | .37 | .03 | .13 |

**BI1, BI2**: Blocking Issues**; EC1, EC2**: Extrapolation Valid for CDSS; **EI**: Extrapolation Issue; **MI3**: Miscellaneous Issue (Absorption phase sample mislabelled as trough)**; Ex.1**: Subset excluding BI1 and BI2; **Ex.2**: Refined subset excluding EC1, EC2, BI1, BI2, EI, MI3.

**Table S5:** Comparative and stratified analysis of intervention outcomes after intervention

|  | All patients (n=60) | | | Ex.1 (n=51) | | | Ex.2 (n=39) | | |
| --- | --- | --- | --- | --- | --- | --- | --- | --- | --- |
| **Category** | **TDM Expert (n)** | **CDSS (n)** | ***P*-value** | **TDM Expert (n)** | **CDSS (n)** | ***P*-value** | **TDM Expert (n)** | **CDSS (n)** | ***P*-value** |
| No Intervention | 39 | 8 | <.001 | 34 | 0 | <.001 | 25 | 0 | <.001 |
| Effective | 10 | 25 | .002 | 8 | 25 | <.001 | 7 | 20 | <.001 |
| Unnecessary | 0 | 7 | .02 | 0 | 7 | .02 | 0 | 7 | .02 |
| Ineffective | 7 | 13 | .11 | 7 | 13 | .11 | 6 | 10 | .29 |
| Detrimental | 1 | 5 | .13 | 0 | 4 | .13 | 0 | 2 | .48 |
| Critical | 3 | 2 | >.99 | 2 | 2 | >.99 | 1 | 0 | >.99 |

# Supplemental Material: Figures

**Figure S1**. Error detection metrics stratified by exclusion scenario. The dot plot compares the performance of the TDM expert (light blue) and the CDSS (dark grey) across sensitivity, specificity, precision, and F1 score and error bars represent the 95% confidence interval (CI_95%_). The analysis was performed on the full standard set (n = 60) and a subset excluding extrapolation cases valid for the CDSS (EC exclusion, i.e. exclusion of extrapolation cases only valid for the CDSS, n = 54).

**Figure S2**. Count of true positives and false negatives stratified by error type. The dot plot details the number of correctly identified issues (True Positives) and missed issues (False Negatives) for the TDM Expert (light blue) and the CDSS (dark grey). The stratification breaks down performance in the whole population (All patients, n=60) and EC exclusion (n=54) sets, as well as by specific error sub-categories defined in Table 2: Extrapolation Valid for CDSS (EC), Blocking Issues (BI), Extrapolation Issues (EI), and Miscellaneous Issues (MI).

**Figure S3.** Distribution of patients within each clinical outcome stratified by different exclusion scenarios. The bar chart compares the clinical outcomes (Within Target, Underexposed, Overexposed) across three study populations: the full set (n = 60), the subset excluding blocking issues (Ex.1, n = 51), and the refined set of exclusion (Ex.2, n = 39) as defined in Table S1. The star (*) and empty circle (°) indicate the statistical significance (*P* <.05) of differences between CDSS and TDM expert or baseline, respectively.

**Figure S4.** Representation of clinical outcomes after CDSS or TDM expert within the whole population (All patients, n=60) and valid cases with the baseline outcome in x-axis and the outcome after intervention in y-axis. Within therapeutic target (W) was defined as 1100–2000 ng/mL for trough steady-state concentration, underexposed (U) and overexposed (O) were defined as below and above these thresholds, respectively [1].

**Figure S5.** Representation of clinical outcomes after CDSS or TDM expert intervention stratified by errors sub-categories, with the baseline outcome in x-axis and the outcome after intervention in y-axis. Within therapeutic target (W) was defined as 1100–2000 ng/mL for trough steady-state concentration, underexposed (U) and overexposed (O) were defined as below and over that threshold, respectively [1].

# References

1.	Goutelle S, Guidi M, Gotta V, Csajka C, Buclin T, Widmer N. From personalized to precision medicine in oncology: A model-based dosing approach to optimize achievement of imatinib target exposure. Pharmaceutics. 2023;15: 1081.
